# Rheumatoid arthritis in the public eye: a study on awareness and knowledge levels in the United Arab Emirates

**DOI:** 10.3389/fpubh.2026.1845147

**Published:** 2026-07-27

**Authors:** Chris Mathew Prince, Adhya Miriam Tom, Sofia Sajid Ali, Jagat Gopinath, Saba Ali, Ashish Sam Enos, Anusha Sreejith

**Affiliations:** 1 College of Medicine, Georgian National University SEU, Tbilisi, Georgia; 2 College of Medicine, Gulf Medical University, Ajman, United Arab Emirates; 3 Department of General Surgery, Thumbay Hospital, Ajman, United Arab Emirates; 4 Department of Community Medicine, Gulf Medical University, Ajman, United Arab Emirates; 5 Faculty of Thumbay Institute of Population Health, Ajman, United Arab Emirates

**Keywords:** attitude, awareness, health literacy, health-seeking behavior, knowledge, patient education, public health, public perception

## Abstract

**Introduction:**

Rheumatoid arthritis (RA) is a chronic autoimmune disease associated with significant morbidity, disability, and reduced quality of life. Despite its clinical importance, public awareness and understanding of RA remain variable across populations. This study aimed to assess the level of awareness, knowledge, attitudes, and information-seeking behavior regarding RA among the general population in the United Arab Emirates (UAE).

**Methods:**

A community-based cross-sectional study was conducted among adults residing in the UAE. Data were collected using a structured, self-administered questionnaire assessing sociodemographic characteristics, awareness, knowledge of symptoms, risk factors, treatment, and attitudes toward RA which was administered in English only. A total of 739 participants were included in the final analysis. Descriptive statistics were used to summarize data, and results were expressed as frequencies and percentages.

**Results:**

Among the participants, 65.4% reported awareness of RA. Knowledge levels were moderate, with 65.2% demonstrating adequate knowledge. A majority correctly identified RA as an autoimmune disease (56%) and recognized its joint involvement (61.4%). However, gaps were observed in symptom recognition, particularly for systemic features such as fever (38.8%) and fatigue (49.7%). Regarding treatment, 64% knew that RA is not completely curable, while knowledge of specific management options varied. In terms of attitudes, 68.2% considered RA a serious condition, and 77.7% would seek medical care for persistent joint symptoms. Most participants (76.7%) preferred obtaining information from the internet, although 20.8% showed reluctance to seek further information.

**Conclusion:**

Although general awareness of RA in the UAE is moderate, significant gaps persist in detailed knowledge, particularly regarding disease mechanisms, systemic involvement, and treatment options. There is a need for targeted educational interventions to improve comprehensive understanding, promote early diagnosis, and enhance disease outcomes.

## Introduction

Rheumatoid arthritis (RA) is one of the most common chronic autoimmune inflammatory diseases, affecting approximately 1% of the global population. However, its prevalence varies significantly across geographic regions, ethnic groups, and socioeconomic settings. While prevalence is estimated to be around 1% in North America, lower rates of approximately 0.2%–0.3% have been reported in populations such as China, Japan, north-west Greece, rural Africa, and Egypt, suggesting the influence of genetic, environmental, and demographic factors on disease distribution. Differences are also observed in public awareness, healthcare-seeking behaviors, and disease-related knowledge across populations (1).

RA is characterized by persistent synovial inflammation leading to joint destruction and functional impairment if left untreated. The disease commonly presents with symmetrical joint pain and prolonged morning stiffness involving the small joints of the hands and feet, with possible progression to larger joints and extra-articular manifestations affecting organs such as the lungs, heart, eyes, and nervous system. The development of RA involves complex interactions between genetic susceptibility and environmental triggers. Diagnosis relies on clinical assessment supported by imaging techniques, including X-ray, magnetic resonance imaging (MRI), and ultrasound, alongside laboratory investigations such as rheumatoid factor (RF), anti-cyclic citrullinated peptide (anti-CCP) antibodies, and inflammatory markers including C-reactive protein (CRP) (2).

Despite its prevalence and significant impact on quality of life, awareness and understanding of RA among the general public remain limited. Previous studies have demonstrated widespread misconceptions regarding disease symptoms, risk factors, systemic complications, and treatment. A study from Netherlands found insufficient public knowledge of rheumatic diseases, although participants expressed interest in receiving additional information (3). Similarly, research from Iraq reported inadequate RA knowledge regardless of educational background, indicating that formal education alone may not address disease awareness gaps (4). In Saudi Arabia, a considerable proportion of participants incorrectly perceived RA as a condition limited to the joints, reflecting poor recognition of its systemic nature (5). Other studies have shown that RA symptoms are often underestimated compared with other chronic diseases, potentially delaying healthcare-seeking behavior and diagnosis (6). Surveys from the UK and China have further identified gaps in awareness of RA epidemiology, disease progression, and management approaches, emphasizing the importance of patient education and improved communication between healthcare providers and communities (7, 8). Furthermore, a survey of 1,050 Arab patients identified significant deficiencies in knowledge about rheumatic diseases and concerns about treatment complications, emphasizing the need for improved patient education and physician-patient communication (9).

Given that early diagnosis and appropriate management are essential to prevent irreversible joint damage and disability, improving public knowledge and awareness of RA remains an important public health priority. Understanding existing knowledge gaps and misconceptions can help guide targeted educational strategies and improve outcomes for individuals affected by this disease (10).

Importantly, despite the global and regional evidence on RA awareness, there remains a lack of large-scale studies assessing public knowledge and awareness of rheumatoid arthritis in the United Arab Emirates (UAE), where the population is highly diverse and multilingual. This represents a significant gap in the literature, particularly in understanding how cultural and demographic diversity may influence health literacy related to autoimmune diseases such as RA. This study aims to evaluate public knowledge, attitudes, and perceptions of RA within the UAE to identify key areas requiring educational and community health interventions.

## Materials and methods

### Study design, population, sample size, and setting

This study employed a community-based cross-sectional design to assess the level of awareness and knowledge regarding rheumatoid arthritis (RA) among the general population in the UAE. The study population comprised adults residing in the UAE from various nationalities. Individuals aged 18 years and above who agreed to participate and provided informed consent were eligible for inclusion. The questionnaire was administered exclusively in English; therefore, only participants who were able to read and understand English were included.

Sample size was calculated using a 95% confidence level (*Z* = 1.96), a margin of error of 5% (*d* = 0.05), and an assumed proportion of 50% (*p* = 0.50). This conservative assumption was applied because the expected prevalence of adequate rheumatoid arthritis awareness/knowledge in the UAE population was unknown at the time of study design and provides the maximum required sample size for a cross-sectional survey. The calculated sample size was 384 participants. After accounting for a potential 10% non-response rate, the minimum required sample size was 422 participants. However, the final number of participants exceeded this estimate due to broader community dissemination of the questionnaire and the use of convenience sampling, which resulted in higher voluntary participation. The study was conducted over a period of 8 months among the general population across the UAE.

This study was designed and reported in accordance with the Strengthening the Reporting of Observational Studies in Epidemiology (STROBE) guidelines for cross-sectional studies. The questionnaire development and reporting procedures were informed by recommendations for survey-based research, including principles outlined in CHERRIES where applicable (11).

### Study instrument and statistical analysis

The questionnaire was developed following a structured process involving literature review of previously published RA awareness and knowledge surveys, identification of relevant domains, and adaptation of previously used concepts. The initial questionnaire included domains assessing sociodemographic characteristics, awareness, disease characteristics, symptoms, risk factors, treatment knowledge, attitudes, and information-seeking behavior. Content validity was assessed through expert review by a clinician with relevant expertise in rheumatology/orthopedics, selected by the authors to evaluate the relevance and appropriateness of the questionnaire items. A pilot test was conducted among five participants from the target population to evaluate clarity, comprehensibility, and completion time. Feedback obtained during pilot testing was used to refine wording and improve questionnaire flow before final administration.

### Data collection procedure

Following Institutional Review Board (IRB) approval, participants were recruited using a convenience sampling strategy from the general adult population residing in the United Arab Emirates. Recruitment was conducted through both online and community-based dissemination. The survey link was distributed via social media platforms (including WhatsApp, Instagram, and community groups), as well as through university networks and personal contacts to ensure broad accessibility across different demographic groups. In addition, eligible individuals encountered in public community settings such as university campuses and healthcare-related environments were invited to participate.

Efforts were made to include participants from different demographic backgrounds; however, recruitment was not restricted by emirate, and participation was voluntary and self-selected. As a result, respondents represented a heterogeneous but non-random sample of the UAE population, and the findings should be interpreted in the context of convenience sampling and potential selection bias. Due to the online dissemination strategy and wide geographic distribution of participants, exact recruitment location and response rate across individual emirates could not be precisely determined.

### Data management and statistical analysis

Data were entered into Microsoft Excel and subsequently imported into SPSS version 29 for analysis. Descriptive statistics were used to summarize participant characteristics and levels of awareness and knowledge regarding rheumatoid arthritis. Results were expressed as frequencies and percentages.

Inferential statistics were applied to determine associations between sociodemographic variables and knowledge levels. The chi-square test was used to assess statistical associations. A *p*-value of <0.05 was considered statistically significant. The knowledge assessment consisted of 15 multiple-choice and closed-ended questions, with each correct response assigned a score of 1 and incorrect responses assigned a score of 0, yielding a total possible score ranging from 0 to 15. Participants scoring more than 8 out of 15 (<53.3%) were categorized as having adequate knowledge, while those scoring 8 or less (≤8/15) were classified as having inadequate knowledge. This cut-off was chosen based on a mid-point threshold of the total score, consistent with approaches used in previous knowledge assessment studies, where scores above 50% are commonly considered indicative of adequate knowledge (12). For example, one of the knowledge questions included was: “Is rheumatoid arthritis an autoimmune disease?” with response options (Yes/No).

## Results

A total of 739 participants were included in the study. From Table 1, the largest proportion of participants aged 30–44 years (33.4%), followed by those aged 45–59 years (30.4%). Participants aged 18–29 years accounted for 22.6%, while those aged ≥60 years represented the smallest group (13.5%). The sample was nearly evenly distributed by sex, with females comprising 50.9% and males 49.1%. Regarding educational attainment, the majority held an undergraduate degree (56.7%), followed by high school education (23.7%) and postgraduate qualifications (19.6%).

**Table 1:** Socio-demographic factors of the participants.

Socio-demographic factors		Frequency	Percentage
Age	18–29 years	167	22.6
	30–44 years	247	33.4
	45–59 years	225	30.4
	Greater than and equal to 60 years	100	13.5
Sex	Female	376	50.9
	Male	363	49.1
Educational level	High school	175	23.7
	Undergraduate degree	419	56.7
	Postgraduate degree	145	19.6

**Figure 1:**
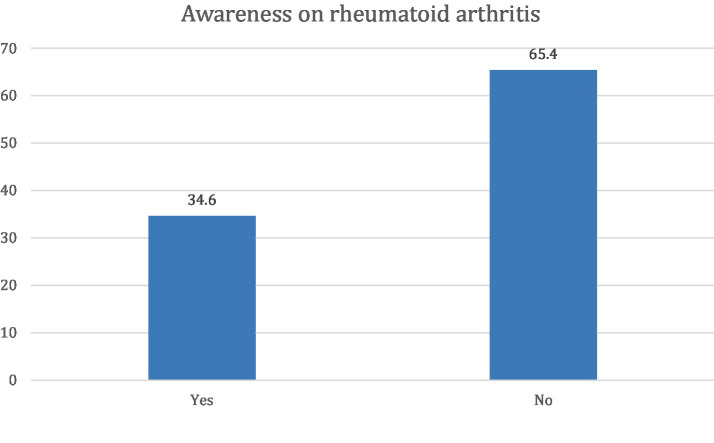
Percentage of participants who are aware of rheumatoid arthritis.

Figure 1 illustrates the awareness of rheumatoid arthritis among the study participants. Out of the total 739 respondents, 483 participants (65.4%) reported that they had heard of rheumatoid arthritis, while 256 participants (34.6%) indicated that they had not heard of the disease.

Table 2 presents the sources of general awareness among participants. Nearly half of the respondents reported having heard about the topic from family or friends (47.2%), while a slightly higher proportion had not (52.8%). Awareness through healthcare professionals was less common, with 37.1% reporting exposure from doctors or nurses compared to 62.9% who had not. Similarly, 49.7% of participants reported learning about the topic through school or university, while 50.3% had not received such information through educational settings.

**Table 2:** Assessment of source of general awareness of rheumatoid arthritis.

Source of general awareness		Frequency	Percentage
Heard from family/friends	Yes	349	47.2
	No	390	52.8
Heard from doctors/nurses	Yes	274	37.1
	No	465	62.9
Learnt from school/university	Yes	367	49.7
	No	372	50.3

Table 3 summarizes participants’ knowledge regarding rheumatoid arthritis. Overall, more than half of respondents correctly identified that rheumatoid arthritis is an autoimmune disease (56.0%) and that it mainly affects the joints (61.4%). Similarly, 63.7% recognized joint pain and 54.4% identified morning stiffness as key symptoms. However, fewer participants correctly identified systemic features, with only 38.8% recognizing fever and 49.7% recognizing fatigue as possible symptoms.

**Table 3:** Assessment of knowledge awareness on rheumatoid arthritis.

Knowledge assessment		Frequency	Percentage
Rheumatoid arthritis is the same as aging bone pain	Yes	338	45.7
	No	401	54.3
Rheumatoid arthritis is an autoimmune disease	Yes	414	56
	No	325	44
Rheumatoid arthritis mainly affects the joints	Yes	454	61.4
	No	285	38.6
Joint pain is a symptom of rheumatoid arthritis	Yes	471	63.7
	No	268	36.3
Morning stiffness is a symptom of rheumatoid arthritis	Yes	402	54.4
	No	337	45.6
Fever is a possible symptom of rheumatoid arthritis	Yes	287	38.8
	No	452	61.2
Fatigue is a symptom of rheumatoid arthritis	Yes	367	49.7
	No	372	50.3
Women are more affected in rheumatoid arthritis	Yes	389	52.6
	No	350	47.4
Painkillers help manage rheumatoid arthritis	Yes	465	62.9
	No	274	37.1
Steroids help manage rheumatoid arthritis	Yes	366	49.5
	No	373	50.5
Physical therapy helps manage rheumatoid arthritis	Yes	418	56.6
	No	321	43.4
Surgery is sometimes used for rheumatoid arthritis	Yes	355	48
	No	384	52
Lifestyle changes can help in rheumatoid arthritis	Yes	440	59.5
	No	299	40.5
If left untreated, can lead to deformity	Yes	484	65.5
	No	255	34.5

In terms of disease perception, 45.7% incorrectly believed rheumatoid arthritis is the same as age-related bone pain. Slightly over half (52.6%) correctly identified that women are more commonly affected. Regarding management, 62.9% recognized the role of painkillers, 56.6% identified physical therapy, and 59.5% acknowledged lifestyle modification as beneficial, while fewer participants recognized steroids (49.5%) or surgery (48.0%) as treatment options. Importantly, 65.5% correctly understood that untreated rheumatoid arthritis can lead to joint deformity.

Table 4 presents participants’ attitudes and beliefs regarding rheumatoid arthritis. Most respondents (68.2%) perceived rheumatoid arthritis as a serious health condition. A higher proportion (77.7%) reported that they would visit a doctor if experiencing persistent joint pain and swelling. In addition, 65.4% believed that individuals with rheumatoid arthritis can live a normal life, while 34.6% did not hold this view.

**Table 4:** Attitude and beliefs of participants on rheumatoid arthritis.

Attitude and beliefs		Frequency	Percentage
Rheumatoid arthritis is a serious health condition	Yes	504	68.2
	No	235	31.8
Visit doctor if there is persistent joint pain and swelling	Yes	574	77.7
	No	165	22.3
People with rheumatoid arthritis can live a normal life	Yes	483	65.4
	No	256	34.6

Table 5 presents participants’ preferred sources of information and gaps regarding rheumatoid arthritis. The majority of respondents indicated that they would seek information from the internet (76.7%) and healthcare providers (69.7%). However, 20.8% reported that they would prefer not to seek additional information about rheumatoid arthritis, while 79.2% expressed willingness to do so.

**Table 5:** Sources of information and gaps of participants on rheumatoid arthritis.

Sources of information and gaps		Frequency	Percentage
Would you go to a healthcare provider to learn more about rheumatoid arthritis?	Yes	515	69.7
	No	224	30.3
Would you look for information on the internet?	Yes	567	76.7
	No	172	23.3
Would you prefer not to seek more information about rheumatoid arthritis?	Yes	154	20.8
	No	585	79.2

**Figure 2:**
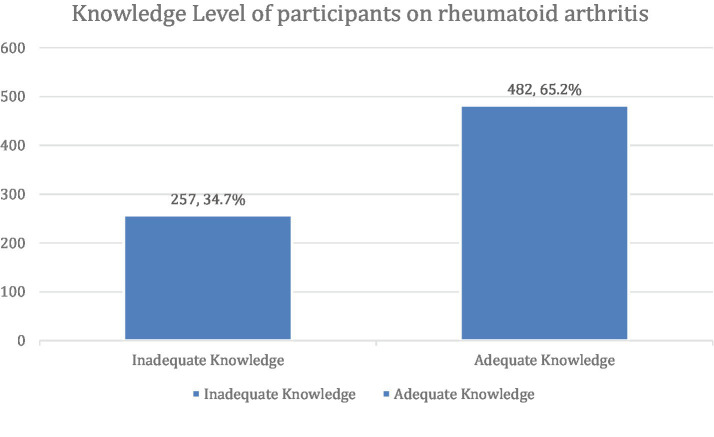
Knowledge levels of participants on rheumatoid arthritis.

From Figure 2, the majority of participants demonstrated adequate knowledge regarding rheumatoid arthritis. Specifically, 482 participants (65.2%) were categorized as having adequate knowledge, while 257 participants (34.7%) exhibited inadequate knowledge.

Table 6 shows the association between socio-demographic factors and participants’ knowledge levels. There was no statistically significant association between age and knowledge level (*p* = 0.337), with adequate knowledge distributed across all age groups. Similarly, sex was not significantly associated with knowledge (*p* = 0.848), with comparable proportions of adequate and inadequate knowledge among males and females. Educational level also showed no significant association with knowledge (*p* = 0.445), although participants with undergraduate degrees constituted the largest proportion of those with adequate knowledge.

**Table 6:** Association between sociodemographic characteristics and knowledge level regarding rheumatoid arthritis among study participants.

Sociodemographic factors		Knowledge		*p*-value
		Inadequate knowledge, *N* (%)	Adequate knowledge, *N* (%)	
Age	18–29 years	64 (8.7)	103 (13.9)	0.337
	30–44 years	83 (11.2)	164 (22.2)	
	45–59 years	82 (11.1)	143 (19.4)	
	Greater than and equal to 60 years	28 (3.8)	72 (9.7)	
Sex	Female	132 (17.9)	244 (33)	0.848
	Male	125 (16.9)	238 (32.2)	
Educational level	High school	64 (8.7)	111 (15)	0.445
	Undergraduate degree	149 (20.2)	270 (36.5)	
	Postgraduate degree	44 (6)	101 (13.7)	

## Discussion

The present study provides a comprehensive assessment of awareness, knowledge, and perceptions of rheumatoid arthritis (RA) among a community-based sample of adults residing in the UAE, offering important insights into public understanding of this chronic condition. While overall awareness appears moderate to good, notable gaps persist across multiple domains, particularly in deeper disease understanding, symptom recognition, and management.

Awareness of RA in this study was reported by 65.4% of participants, comparable to findings from Lebanon, where nearly two-thirds of individuals had heard of the disease (13). However, consistent with findings from Netherlands, gaps in understanding remain evident, particularly regarding disease prevalence and differentiation from other rheumatic conditions (2). Sources of information were also similar across regions, with informal sources such as family and friends being the most common, while healthcare professionals contributed less frequently (13). Although formal education played a role in awareness, its impact remains insufficient. In contrast, studies from Egypt have demonstrated considerably lower knowledge levels, with most participants categorized as having poor to average knowledge (1), highlighting regional disparities.

With respect to disease understanding, only a proportion of participants demonstrated accurate knowledge of RA as an autoimmune condition. While studies from Egypt and Saudi Arabia reported low awareness of its autoimmune nature (14.2% and 26.6%, respectively) (14, 15), the present study showed relatively higher awareness (56%), suggesting some improvement in baseline knowledge. Similarly, 61.4% of participants correctly identified joint involvement, aligning with findings from other regional studies that reported better recognition of joint-related inflammation (10). Despite this, misconceptions persist, indicating that fundamental disease concepts are not yet universally understood.

In terms of symptom recognition, participants showed reasonable awareness of common joint-related manifestations, with 63.7% identifying joint pain and 54.4% recognizing morning stiffness. These findings are broadly comparable to studies from Saudi Arabia, where higher proportions identified key symptoms such as fatigue and prolonged morning stiffness (10). However, awareness of systemic features, including fatigue and fever, remained limited in the present study, reflecting an incomplete understanding of the multisystem nature of RA.

Knowledge of risk factors demonstrated moderate awareness, particularly regarding sex predisposition. While earlier studies reported low recognition of female predominance (28.6%) (16), the current study found that 52.6% of participants correctly identified this association, suggesting gradual improvement in public awareness of epidemiological patterns.

Understanding of RA management was variable. Although a majority of participants recognized the role of pain relief and non-pharmacological interventions such as physical therapy, awareness of specific treatment modalities, including steroids and surgical options, was less consistent. Compared to studies from Egypt, where a high proportion of participants were aware that RA requires lifelong treatment (14), the present findings indicate that while some aspects of disease chronicity are understood, detailed knowledge of treatment strategies remains limited.

Encouragingly, attitudes toward RA were generally positive. Most participants recognized RA as a serious condition and acknowledged the importance of seeking medical care for persistent joint symptoms, consistent with findings from Saudi Arabia (15). Additionally, a majority believed that individuals with RA can lead normal lives, which aligns with current evidence demonstrating improved outcomes with early diagnosis and appropriate treatment (17). However, global reports continue to emphasize that RA can lead to significant disability if not adequately managed (18), underscoring the importance of accurate and balanced public perception.

Regarding information-seeking behavior, the study highlights a growing reliance on digital platforms, with more participants preferring to seek information online than from healthcare providers. This trend mirrors global patterns (18), but also raises concerns about the quality and reliability of online health information. Notably, a subset of participants expressed reluctance to seek further information, reflecting an important gap that may contribute to delayed diagnosis and suboptimal disease management (18).

With respect to age, our findings are consistent with previous population-based studies conducted in Saudi Arabia, which demonstrated that demographic variables such as age did not significantly influence awareness or knowledge of rheumatoid arthritis (RA) (10). This suggests that exposure to RA-related information may be uniformly limited across different age groups, highlighting a widespread gap in public awareness.

Similarly, our findings regarding educational level are in agreement with a study conducted in Al-Qassim, Saudi Arabia, which reported no statistically significant association between level of education and RA knowledge (16). In our study, although participants with an undergraduate degree constituted the largest proportion of those with adequate knowledge (36.5%), followed by postgraduate (13.7%) and high school education (15%), these differences were not statistically significant. This indicates that higher formal education does not necessarily translate into better disease-specific awareness, emphasizing the need for targeted health education interventions.

In contrast to some regional findings, a study from Saudi Arabia reported that female sex and higher family income were significantly associated with better RA knowledge (OR = 3.65; 95% CI: 0.06–12.62) (19). However, our results did not demonstrate a similar trend, as the distribution of adequate knowledge was comparable between females (33%) and males (32.2%), with no statistically significant sex-based difference. Likewise, no significant association was observed between educational level and knowledge in our cohort.

Overall, these findings suggest that while certain studies have identified demographic predictors of RA awareness, knowledge levels in our population appear to be relatively uniform across sociodemographic groups. This further reinforces the importance of implementing broad, population-wide awareness strategies rather than focusing solely on specific demographic subgroups.

This study has limitations. The cross-sectional design prevents establishing causal relationships between sociodemographic factors and knowledge levels. The questionnaire was reviewed by an orthopedic specialist without rheumatologist input, which may have limited clinical validation of RA-specific content. Self-reported data may be subject to response bias. Additionally, the English-only survey may have excluded non-English-speaking populations in the multilingual UAE context, limiting generalizability. Participants with prior RA diagnoses were not excluded, which may have influenced knowledge scores. Finally, the absence of effect size measures (e.g., Cramér’s V) limits interpretation of the strength of observed associations.

In conclusion, this study represents one of the few comprehensive assessments of rheumatoid arthritis awareness in the UAE and highlights a critical gap between general awareness and in-depth understanding. While encouraging trends are observed in symptom recognition and health-seeking attitudes, significant deficiencies remain in knowledge of disease mechanisms, systemic features, and treatment options.

These findings suggest the potential value of culturally appropriate and multilingual public health education strategies to address gaps in RA knowledge and improve awareness of early symptoms and treatment pathways. Such interventions should include multilingual awareness campaigns (Arabic, English, Urdu, and Malayalam) delivered through social media platforms, development of school- and university-based educational modules on autoimmune diseases, and integration of simple visual posters and awareness materials in primary healthcare centers and waiting areas. In addition, engagement of community influencers and primary care physicians in delivering brief educational messages during routine consultations may further enhance public understanding. Strengthening public awareness through these practical and culturally inclusive strategies is essential to promote early diagnosis, improve treatment outcomes, and ultimately reduce the long-term burden of rheumatoid arthritis in the region.

## Data Availability

The original contributions presented in the study are included in the article/Supplementary material, further inquiries can be directed to the corresponding author.

## References

[1] El Saman AM Mohamed ER Khalifa A Meghezel Z Radwan A. Evaluation of awareness, knowledge and attitudes regarding common rheumatic diseases (rheumatoid arthritis and systemic lupus erythematous) in Sohag governorate. Egypt J Community Med. (2020) 38:25–36. doi: 10.21608/ejcm.2020.89887

[2] Trivedi J. Clinical presentation and diagnosis of rheumatoid arthritis. Ann Clin Med Case Rep. (2024) 13:1–7.

[3] Van Der Wardt EM Taal E Rasker JJ. The general public's knowledge and perceptions about rheumatic diseases. Ann Rheum Dis. (2000) 59:32–8. doi: 10.1136/ard.59.1.32, 10627424 PMC1752978

[4] Salman S SAlnuaimi A Lateef NA Kadhum R. Assessment of knowledge and attitude in a sample of patients with rheumatoid arthritis and its association with disease activity and severity: a cross-sectional study. Open J Rheumatol Autoimmune Dis. (2014) 4:226–34. doi: 10.4236/ojra.2014.44031

[5] Alzubaidi HA Taha MF Alfaqih AN Hafiz W Alhayli AA Alfaqih HM . Knowledge and awareness of rheumatoid arthritis among the population of Al Qunfudhah, Saudi Arabia: a cross-sectional study. Ann Rheumatol Autoimmun. (2024) 4:35–40. doi: 10.4103/ara.ara_11_24

[6] Simons G Belcher J Morton C Kumar K Falahee M Mallen CD . Symptom recognition and perceived urgency of help-seeking for rheumatoid arthritis and other diseases in the general public: a mixed method approach. Arthritis Care Res. (2017) 69:633–41. doi: 10.1002/acr.22979, 27389847 PMC5518890

[7] National Rheumatoid arthritis Society (NRAS). Breaking Down Barriers: Rheumatoid Arthritis and Public Awareness. Maidenhead: NRAS (2013).

[8] Li X Liu J Yu J Dong L. Knowledge, attitudes, and practices of dietary management among patients with rheumatoid arthritis in China. Front Public Health. (2024) 12:1490189. doi: 10.3389/fpubh.2024.1490189, 39583079 PMC11582067

[9] Alnaqbi KA Alaswad M Alasfour S. Patient perspectives on rheumatic and musculoskeletal diseases: insights from a large-scale survey. Clin Rheumatol. (2025) 44:1457–66. doi: 10.1007/s10067-025-07388-x, 40047988

[10] Alrashidi FF Alzahim MK Alluhaibi ST Alansari AA Aljarboua FA Alkhurayyif AI . Public awareness of rheumatoid arthritis as a genetically influenced autoimmune disease: a population-based study in Riyadh, Saudi Arabia. Genet Mol Res. (2025) 24:1–2. doi: 10.4238/smg34x86

[11] Zimba O Gasparyan AY. Designing, conducting, and reporting survey studies: a primer for researchers. J Korean Med Sci. (2023) 38. doi: 10.3346/jkms.2023.38.e403, 38084027 PMC10713437

[12] Albiss L Muflih S Hijazi B Alshogran OY Al-Qerem W Khurmah MA . Disease knowledge and quality of life among rheumatoid arthritis patients: a cross-sectional study. BMC Rheumatol. (2025) 9:77. doi: 10.1186/s41927-025-00523-w, 40597453 PMC12220810

[13] Menassa J Bou Nassar D El Naboulsi F El Naggar E Sunna N Ghoubar M. Public awareness of rheumatoid arthritis and ankylosing spondylitis in Lebanon. Open Rheumatol J. (2022) 16. doi: 10.2174/18743129-v16-e221130-2022-7

[14] El-Zorkany BA Abdelrahman W Lobna AM El-Ghobashy NE Eissa M. Evaluation of rheumatoid arthritis knowledge among a cohort of 1004 healthy Egyptians. Med J Cairo Univ. (2023) 91:225. doi: 10.21608/mjcu.2023.307506

[15] Al-Mehmadi BA Alelaiwi MM Alnumayr HS Alghamdi BS Alomari BA Alzahrani HS. Knowledge of common symptoms of rheumatic diseases and causes of delayed diagnosis in Saudi Arabia. Patient Prefer Adherence. (2024) 18:635–47. doi: 10.2147/PPA.S448999, 38476592 PMC10929651

[16] Alrashdi MN Alrasheedi SM Alkhdairi A Almutairi KO Almutairi MA Alharbi AF . Impact of mass media on the general population's knowledge and attitudes toward rheumatoid arthritis in Qassim, Saudi Arabia. Cureus. (2022) 14. doi: 10.7759/cureus.31079, 36475162 PMC9719607

[17] Mayo Clinic Rheumatoid Arthritis—Symptoms and Causes. Rochester, Minnesota, USA: Mayo Clinic (2025). Available online at: https://www.mayoclinic.org/diseases-conditions/rheumatoid-arthritis/symptoms-causes/syc-2035364 (accessed February 16, 2026).

[18] World Health Organization Rheumatoid Arthritis. Geneva, Switzerland: World Health Organization (2023). Available online at: https://www.who.int/news-room/fact-sheets/detail/rheumatoid-arthritis (accessed February 16, 2026).

[19] AlMalki HH Bedywi RM Alzayd HA AlQahtani AM AlOmair MM. Assessment of disease-related knowledge among patients with rheumatoid arthritis in Aseer region, Saudi Arabia: a cross-sectional study. Ann Rheumatol Autoimmun. (2025) 5:54–60. doi: 10.4103/ara.ara_34_24

