# Supplementary material for: Rheumatoid arthritis in the public eye: a study on awareness and knowledge levels in the United Arab Emirates
Source: Front Public Health. 2026 Jul 27;14:1845147. doi: 10.3389/fpubh.2026.1845147 (PMC13454645; doi:10.3389/fpubh.2026.1845147)
Supplement: Supplementary file 1 [file Table_1.DOCX]

**Rheumatoid Arthritis in the Public Eye: A Study on Awareness and Knowledge Levels in UAE**

1. Age:
2. Gender:
3. Educational level:
4. Employment status:
5. **HAVE YOU HEARD OF RHEUMATOID ARTHRITIS:**

| **General Awareness** | Yes | No |
| --- | --- | --- |
| Did a family member or friend first tell you about rheumatoid arthritis? |  |  |
| Did you first learn about rheumatoid arthritis from a doctor or nurse? |  |  |
| Did you first learn about it in school or university? |  |  |

| **Knowledge Assessment** | Yes | No |
| --- | --- | --- |
| Rheumatoid arthritis is the same as general bone pain from aging. |  |  |
| Rheumatoid arthritis is an autoimmune disease. |  |  |
| Rheumatoid arthritis mainly affects the joints |  |  |
| Do you think joint pain is a symptom of rheumatoid arthritis? |  |  |
| Is morning stiffness a common symptom? |  |  |
| Is fever a possible symptom? |  |  |
| Is fatigue a symptom of rheumatoid arthritis? |  |  |
| Are women more commonly affected than men? |  |  |
| Can rheumatoid arthritis be completely cured? |  |  |
| Can painkillers help manage rheumatoid arthritis? |  |  |
| Can steroids be used in treatment? |  |  |
| Can physical therapy help patients with rheumatoid arthritis? |  |  |
| Is surgery sometimes used to manage rheumatoid arthritis? |  |  |
| Can lifestyle changes help manage rheumatoid arthritis? |  |  |
| If untreated, can rheumatoid arthritis lead to joint deformity or disability? |  |  |

| **Attitude and Beliefs** | Yes | No |
| --- | --- | --- |
| Do you think rheumatoid arthritis is a serious health condition? |  |  |
| Would you visit a doctor if you had persistent joint pain and swelling? |  |  |
| Do you believe people with rheumatoid arthritis can live a normal life with proper treatment? |  |  |

| **Sources of Information and Gaps** | Yes | No |
| --- | --- | --- |
| Would you go to a healthcare provider to learn more about rheumatoid arthritis? |  |  |
| Would you look for information on the internet? |  |  |
| Would you prefer not to seek more information about rheumatoid arthritis? |  |  |
